# A simulation-based policy analysis of anticipatory action for cholera outbreaks, Democratic Republic of the Congo

**DOI:** 10.2471/BLT.25.293226

**Published:** 2025-09-03

**Authors:** Pei Shan Loo, Jefferson K Rajah, Hugo Jose Herrera de Leon, Birgit Kopainsky, Leonardo Milano

**Affiliations:** a Swiss Tropical and Public Health Institute, Department of Epidemiology and Public Health, Kreuzstrasse 2, Allschwil, 4123, Switzerland.; b System Dynamics Group, University of Bergen, Bergen, Norway.; c The Centre for Humanitarian Data, United Nations Office for the Coordination of Humanitarian Affairs, The Hague, Kingdom of the Netherlands.

## Abstract

**Objective:**

To explore the effect of anticipatory action on outcomes during a cholera outbreak in a hypothetical health zone in the Democratic Republic of the Congo by means of a cholera response model.

**Methods:**

Using a system dynamics approach, we developed a cholera response model for the Democratic Republic of the Congo on the basis of a published cholera response simulation model for Yemen. The model evaluated four intervention scenarios: (i) existing responses to cholera outbreaks; (ii) anticipatory action (that is, immediate interventions); (iii) anticipatory action plus one vaccine dose; and (iv) anticipatory action plus two vaccine doses.

**Findings:**

The model showed that immediate interventions can function as an essential bridge to comprehensive vaccination, particularly in resource-constrained settings where timely coordination is crucial. Moreover, anticipatory action can reduce the total number of cholera cases. However, booster vaccinations are crucial for preventing subsequent waves of infection due to waning immunity following single-dose vaccination.

**Conclusion:**

Anticipatory action can enhance cholera outbreak management in low-resource settings by facilitating synergy between immediate and long-term interventions. The timing and coordination of interventions and the use of booster doses to prevent disease resurgence are all important. Dynamic models are useful for simulating outbreaks and can foster proactive, evidence-based public health planning, thereby supporting the shift from reactive to anticipatory strategies in alignment with the Global Task Force on Cholera Control’s 2030 cholera roadmap. Continuous refinement of the model with real-world data will enhance its global applicability and help advance effective disease control strategies.

## Résumé

**Objectif:**

Étudier l’effet de mesures anticipatives sur les résultats lors d’une épidémie hypothétique de choléra en République démocratique du Congo à l’aide d’un modèle de lutte contre le choléra.

**Méthodes:**

À l’aide d’une approche dynamique des systèmes, nous avons mis au point un modèle de lutte contre le choléra pour la République démocratique du Congo sur la base d’un modèle de simulation de lutte contre le choléra, publié pour le Yémen. Ce modèle a évalué quatre scénarios d’intervention: i) l’action existante contre les épidémies de choléra; ii) les mesures anticipatives (c’est-à-dire les interventions immédiates); iii) les mesures anticipatives ainsi qu’une dose de vaccin; et iv) les mesures anticipatives ainsi que deux doses de vaccin.

**Résultats:**

Le modèle a mis en évidence que des interventions immédiates peuvent contribuer de manière essentielle à une vaccination complète, en particulier dans des contextes de ressources limitées et de nécessité cruciale d’une coordination rapide. De plus, les mesures anticipatives permettent de réduire le nombre total de cas de choléra. Cependant, les vaccins de rappel sont essentiels pour prévenir les vagues ultérieures d’infection dues au déclin de l’immunité après une vaccination à dose unique.

**Conclusion:**

Des mesures anticipées permettent d’améliorer la gestion des épidémies de choléra dans les milieux à faibles ressources en facilitant la synergie entre les interventions immédiates et à long terme. La rapidité et la coordination des interventions ainsi que le recours à des doses de rappel pour prévenir la résurgence de la maladie sont tous importants. Les modèles dynamiques sont utiles pour simuler les épidémies et favorisent une planification proactive et fondée sur des données scientifiques en matière de santé publique, soutenant ainsi la transition de stratégies réactives à des stratégies anticipatives, conformément à la feuille de route 2030 du Groupe de travail mondial sur la lutte contre le choléra (Global Task Force on Cholera Control). L’amélioration continue de ce modèle à l’aide de données réelles renforcera son applicabilité à l’échelle mondiale et contribuera à faire progresser les stratégies efficaces de lutte contre la maladie.

## Resumen

**Objetivo:**

Explorar el efecto de la acción anticipatoria en los resultados durante un brote de cólera en una zona de salud hipotética de la República Democrática del Congo mediante un modelo de respuesta al cólera.

**Métodos:**

Con un enfoque de dinámica de sistemas, se desarrolló un modelo de respuesta al cólera para la República Democrática del Congo a partir de un modelo de simulación de respuesta al cólera previamente publicado para el Yemen. El modelo evaluó cuatro escenarios de intervención: (I) respuestas existentes a brotes de cólera; (ii) acción anticipatoria (es decir, intervenciones inmediatas); (iii) acción anticipatoria más una dosis de vacuna; y (iv) acción anticipatoria más dos dosis de vacuna.

**Resultados:**

El modelo mostró que las intervenciones inmediatas pueden funcionar como un puente esencial hacia la vacunación integral, particularmente en entornos con recursos limitados donde la coordinación oportuna resulta crucial. Además, la acción anticipatoria puede reducir el número total de casos de cólera. Sin embargo, las dosis de refuerzo son fundamentales para prevenir oleadas posteriores de infección debido a la disminución de la inmunidad tras la vacunación con una sola dosis.

**Conclusión:**

La acción anticipatoria puede mejorar la gestión de los brotes de cólera en entornos con recursos limitados al facilitar la sinergia entre intervenciones inmediatas y a largo plazo. El momento y la coordinación de las intervenciones, así como el uso de dosis de refuerzo para prevenir el resurgimiento de la enfermedad, resultan determinantes. Los modelos dinámicos son útiles para simular brotes y pueden fomentar una planificación de salud pública proactiva y basada en la evidencia, apoyando así la transición de estrategias reactivas a anticipatorias en consonancia con la hoja de ruta 2030 para el cólera del Grupo Especial Mundial sobre el Control del Cólera. El perfeccionamiento continuo del modelo con datos del mundo real reforzará su aplicabilidad global y contribuirá al avance de estrategias eficaces de control de la enfermedad.

## ملخص

الغرض استكشاف تأثير الإجراء الاستباقي على النتائج خلال تفشي الكوليرا في منطقة صحية افتراضية بجمهورية الكونغو الديمقراطية، وذلك باستخدام نموذج الاستجابة للكوليرا.

الطريقة باستخدام أسلوب ديناميكيات النظام، قمنا بتطوير نموذج استجابة للكوليرا لجمهورية الكونغو الديمقراطية، استنادًا إلى نموذج محاكاة منشور للاستجابة للكوليرا في اليمن. قام النموذج بتقييم أربعة سيناريوهات للتدخل: (1) الاستجابات الحالية لحالات تفشي الكوليرا؛ و(2) الإجراء الاستباقي (أي التدخلات الفورية)؛ و(3) الإجراء الاستباقي بالإضافة إلى جرعة لقاح واحدة؛ و(4) الإجراء الاستباقي بالإضافة إلى جرعتين من اللقاح.

النتائج أظهر النموذج أن التدخلات الفورية يمكنها أن تكون بمثابة وسيلة أساسية للتطعيم الشامل، وخاصة في البيئات محدودة الموارد، حيث يكون التنسيق في الوقت المناسب أمرًا بالغ الأهمية. بالإضافة إلى ذلك، يمكن للإجراء الاستباقي أن يقلل من العدد الإجمالي لحالات الإصابة بالكوليرا. ومع ذلك، تُعدّ التطعيمات المُعزّزة بالغة الأهمية للوقاية من موجات العدوى اللاحقة الناتجة عن ضعف المناعة بعد التطعيم بجرعة واحدة.

الاستنتاج يُمكن للإجراء الاستباقي أن يُحسّن من إدارة تفشي الكوليرا في البيئات منخفضة الموارد، وذلك من خلال تسهيل التعاون بين التدخلات الفورية والتدخلات طويلة الأجل. يُعدّ توقيت وتنسيق التدخلات، واستخدام الجرعات المُعزّزة لمنع عودة ظهور المرض، من الأمور المهمة. وتُعدّ النماذج الديناميكية مفيدة لمحاكاة حالات تفشي المرض، ويمكنها تعزيز التخطيط الاستباقي للصحة العامة القائم على الأدلة، مما يدعم التحول من الاستراتيجيات التفاعلية إلى الاستراتيجيات الاستباقية، بما يتماشى مع خارطة طريق الكوليرا لعام 2030 التي وضعها فريق العمل العالمي لمكافحة الكوليرا. وسيؤدي التطوير المستمر للنموذج باستخدام بيانات واقعية، لتعزيز قابليته للتطبيق عالميًا، ويُساعد في تطوير استراتيجيات فعّالة لمكافحة المرض.

## 摘要

**目的:**

旨在通过一个霍乱应对模型探讨前瞻性行动对刚果民主共和国某个假设性卫生区霍乱爆发期间疾病防治结果的影响。

**方法:**

根据也门已公布的霍乱应对模拟模型,我们运用系统动力学方法为刚果民主共和国构建了一个霍乱应对模型。该模型评估了以下四种干预情景:(i) 现有的霍乱爆发应对措施;(ii) 前瞻性行动(即即时干预措施);(iii) 前瞻性行动与单剂疫苗接种;以及 (iv) 前瞻性行动与两剂疫苗接种。

**结果:**

该模型表明,即时干预措施可能是实现全面疫苗接种的一种必要手段,特别是在资源受限且及时协调工作至关重要的地区。此外,前瞻性行动有助于减少霍乱病例总数。但是,患者在接种单剂疫苗后会出现免疫力下降的情况,因此加强针接种是防止后续出现感染浪潮的至关重要举措。

**结论:**

前瞻性行动可促进即时干预措施和长期干预措施之间的协同作用,从而有助于加强对资源匮乏地区霍乱爆发问题的管理。干预措施的实施时机和协调实施以及加强针的推行都是防止疾病复发关键要素。动态模型非常适合模拟霍乱爆发,且有助于推动前瞻性循证公共卫生规划,从而可实现从反应式战略到前瞻性战略的转变,这与全球霍乱控制专题小组发布的《2030 年霍乱防治路线图》相契合。根据真实世界数据对该模型进行持续改进,这将提高其全球适用性并有助于推行有效的疾病防治战略。

## Резюме

**Цель:**

Изучить влияние упреждающих действий на исход вспышки холеры в гипотетической зоне здравоохранения в Демократической Республике Конго с применением модели реагирования на холеру.

**Методы:**

Авторы использовали системный динамический подход для разработки модели реагирования на вспышку холеры в Демократической Республике Конго на основе опубликованной имитационной модели реагирования на холеру для Йемена. В модели оценивались четыре сценария реагирования: (і) существующие меры реагирования на вспышку холеры; (іі) упреждающие действия (то есть неотложные вмешательства); (ііі) упреждающие действия плюс одна доза вакцины; (iv) упреждающие действия плюс две дозы вакцины.

**Результаты:**

Модель показала, что неотложные вмешательства могут служить важнейшим переходным этапом на пути достижения всеобщей вакцинации, особенно в условиях ограниченных ресурсов, когда своевременная координация действий имеет решающее значение. Более того, упреждающие действия могут снизить количество заболевших холерой. Однако бустерная вакцинация является критически важной для предотвращения последующих волн инфекции, поскольку иммунитет после первой дозы вакцины со временем ослабевает.

**Вывод:**

Упреждающие действия могут улучшить ход борьбы со вспышкой холеры в условиях ограниченных ресурсов, так как они содействуют синергии между неотложными и долговременными вмешательствами. В этом случае важны своевременность и координация вмешательств и использование бустерных доз для предотвращения возврата заболевания. Динамические модели полезны для имитирования вспышек и могут содействовать проактивному планированию общественного здравоохранения с опорой на доказательную базу, тем самым способствуя переходу от стратегий реагирования к стратегиям упреждения в соответствии с планом действий Глобальной целевой группы по борьбе с холерой на период до 2030 года. Постоянное уточнение модели с использованием фактических данных улучшит ее глобальную применимость и поможет совершенствовать эффективные стратегии контроля заболеваний.

## Introduction

Cholera, caused by the *Vibrio cholerae* bacterium, remains a global health challenge, particularly in regions with inadequate water, sanitation and hygiene infrastructure.^1^^,^^2^ The disease causes severe symptoms such as watery diarrhoea and dehydration, which can be fatal without timely treatment. Although proper health care reduces the case fatality rate to below 1%, untreated cholera can result in a mortality rate as high as 70%.^3^^,^^4^

The Democratic Republic of the Congo bears a disproportionate cholera burden. The country accounted for up to 14% of the estimated 1.34 to 4.01 million cases that occurred globally each year between 2008 and 2012.^5^^,^^6^ In 2017, the country experienced one of its worst outbreaks, with over 53 000 cases and 1145 deaths.^5^ Despite ongoing efforts, a resurgence in 2022 highlighted the persistent challenge of cholera control.^7^

Anticipatory action offers a proactive alternative to traditional reactive responses to events. This approach involves implementing pre-emptive measures to mitigate the impact of crises.^8^ Humanitarian agencies such as the International Federation of Red Cross and Red Crescent Societies, the United Nations Children's Fund and the World Health Organization (WHO) have increasingly adopted anticipatory action, with an emphasis on early warning systems, preplanned interventions and pre-arranged funding aimed at reducing the disease burden.^9^^,^^10^ This approach aligns with the Global Task Force on Cholera Control’s roadmap for eliminating cholera as a public health threat by 2030.^11^ Although anticipatory approaches have been explored in broader disasters,^10^^,^^12^ their inclusion in models of cholera interventions remains limited, despite the need for proactive strategies in high-burden settings like the Democratic Republic of the Congo.

Most previous cholera models have focused on reactive strategies. Researchers have analysed reactive vaccination and localized interventions during outbreaks,^13^^,^^14^ or discussed cholera transmission models that highlighted challenges such as parameter uncertainty and the need for a proactive approach.^15^ In a modelling study of a cholera outbreak in Yemen,^16^ researchers showed that early intervention could have prevented up to 40% of deaths compared with interventions one year later at the end of the epidemic curve. However, their study did not incorporate anticipatory action.

The aim of our study was to adapt and expand the Yemen cholera response model^16^ to investigate the effect of anticipatory action on cholera outcomes in the Democratic Republic of the Congo. The model simulates the effect of different intervention scenarios and provides an insight into the use of proactive public health strategies in low-resource settings.

## Methods

Our study used a system dynamics model to simulate the effect of cholera interventions in the Democratic Republic of the Congo. System dynamics modelling aims to provide an understanding of how complex systems behave over time by analysing feedback loops and causal relationships, which makes it particularly useful for health policy planning in low-resource settings.^17^^,^^18^ The model also provides a framework for investigating intervention scenarios, even when data availability is limited.^19^

We adapted a published Yemen cholera response model by building on the model’s susceptible–infected–recovered–susceptible framework and by integrating water, sanitation and hygiene measures and health-care and vaccination interventions. In the susceptible–infected–recovered–susceptible framework, individuals transition between the states of being susceptible to infection, infected, recovered and susceptible again when they eventually experience waning immunity after recovery. To reflect local conditions in the Democratic Republic of the Congo, the model incorporated data for Nyiragongo, North Kivu, where there have been recurring cholera outbreaks and where public health resources are constrained.^6^^,^^20^ A detailed description of the original model structure is available in the original article.^16^

To isolate the systemic impact of interventions and to allow generalization to other settings with a high disease burden, the model used a hypothetical health-zone framework. This approach avoids confounding by context-specific factors such as conflict or population displacement (for example, internally displaced persons or refugee camps) and ensures the analysis focuses on broader population dynamics. Despite being hypothetical, the model’s parameters were based on real demographic and epidemiological data from North Kivu, including population statistics and information on documented interventions and resource availability obtained from WHO reports, publications of the Democratic Republic of the Congo’s health ministry and peer-reviewed studies (online repository).^23^ Where data were incomplete, we incorporated plausible ranges of parameters into sensitivity analyses to estimate uncertainty and to ensure realistic and scalable insights could be derived for intervention planning.

### Model data

Our cholera response model relied on three main data sources: (i) structural data, which were retained from the Yemen cholera response model’s susceptible–infected–recovered–susceptible framework and which addressed general cholera transmission dynamics; (ii) epidemiological data, which were derived from the global and regional literature and which covered infection duration, vaccine immunity and other biological constants; and (iii) intervention data augmented by region-specific water, sanitation and hygiene data and health-care response data that reflected conditions in Nyiragongo.

As the primary aim of the study was to assess early outbreak responses at the population level, we did not disaggregate data by age, sex or gender. Moreover, there were data limitations and a need for model simplicity. We followed Sex and Gender Equity in Research guidelines where possible, and remained aware of the importance of sex and gender in health research throughout our analysis.^21^

The model simulated cholera interventions over a 3-year period from 1 January 2022 to 1 January 2025. We used Stella Architect version 3.4.1 (isee systems, Lebanon, United States of America) for the simulations and Euler’s integration method was applied with a time step of 0.25 days.^22^ We made adaptations to ensure the model reflected the local context in North Kivu: (i) case-area targeted interventions were introduced to simulate localized response strategies; and (ii) sewage treatment interventions were excluded due to a lack of sewage treatment facilities in the area.

### Cholera dynamics

**Fig. 1:**
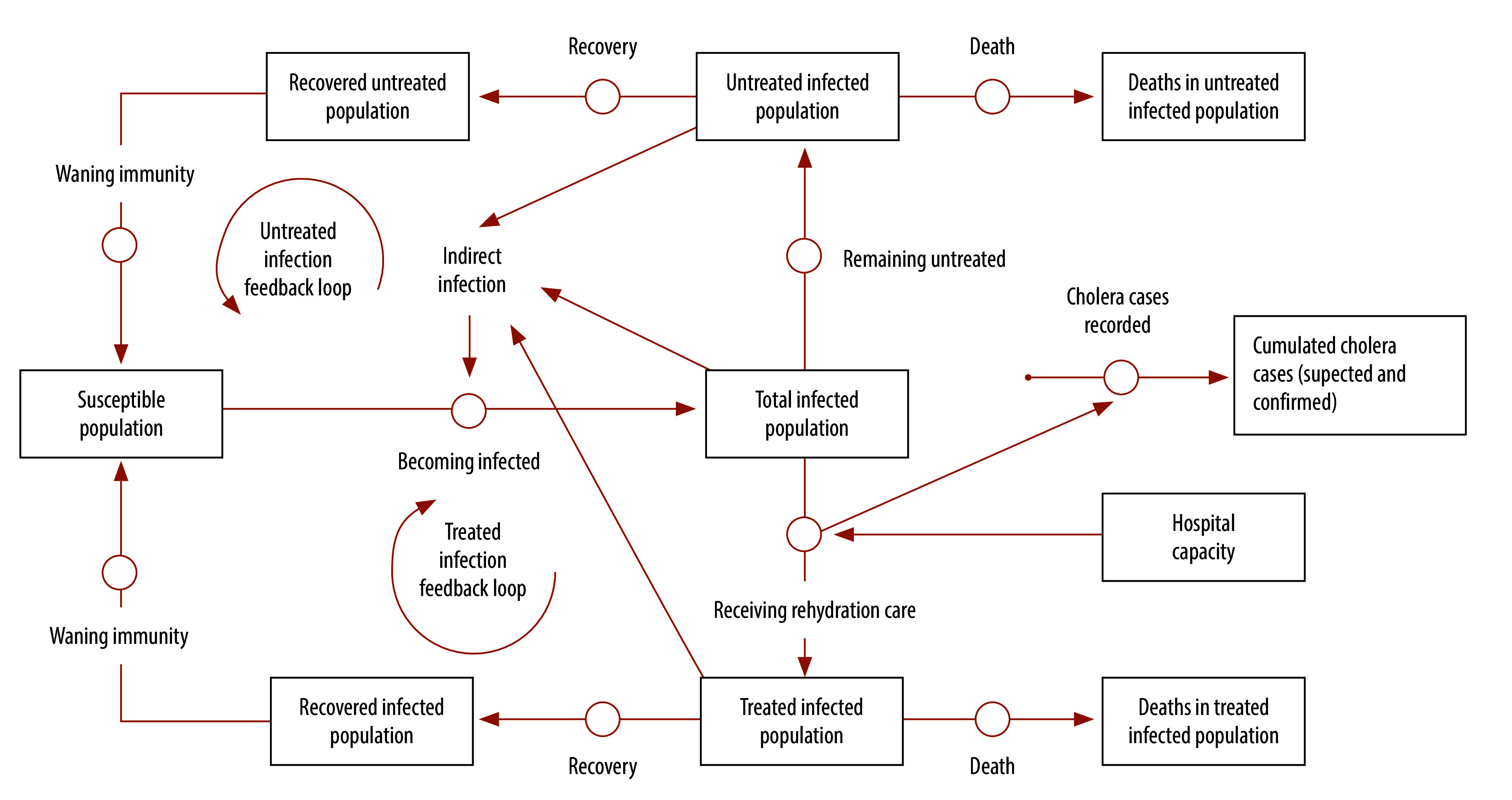
Simplified diagram of infection processes and interventions in the cholera response model before declaration of a cholera outbreak, modelling study of the effect of anticipatory action on cholera outbreaks in the Democratic Republic of the Congo

Notes: The cholera response model was based on a system dynamics model devised using data from a published model for Yemen.^16^ Indirect infection refers to the spread of cholera to susceptible individuals through contact with water that has been contaminated by individuals who are already infected.

Fig. 1 shows a simplified version of the adapted cholera response model for the Democratic Republic of the Congo based on the susceptible–infected–recovered–susceptible framework. Detailed model descriptions and parameter values are provided in the online repository.^23^

In the model, cholera transmission takes place primarily through contaminated water (that is, indirect infection), which is driven by bacterial shedding from infected individuals and an inadequate water, sanitation and hygiene infrastructure. In the Democratic Republic of the Congo, only an estimated 52% of the population has access to basic drinking water and over 7 million people practice open defecation, which underscores the urgent need for water, sanitation and hygiene interventions.^24^^,^^25^

The infected population was divided into people who remain untreated and those who receive care, with two associated infection feedback loops (Fig. 1). In the treated infection feedback loop, individuals, who were typically managed at cholera treatment centres, did not contribute to environmental contamination as their waste was disinfected, thereby reducing the spread of *Vibrio cholerae*.^3^ In the untreated infection feedback loop, individuals continued to shed bacteria into the environment, thereby driving indirect infection. In one study in Yemen,^26^ only an estimated 32% of people with suspected cholera visited a cholera treatment centre on the day of symptom onset,^50^ which delayed treatment and increased the risk of the disease spreading.

The cholera recovery time varies: asymptomatic cases recover within 5 days and symptomatic cases recover within 9 days.^13^^,^^27^ Severely infected individuals either recover with immunity or die during this time period.

The duration of immunity also varies: in symptomatic cases, immunity typically lasts 3 years; whereas in asymptomatic cases, immunity may last only 3 to 12 months.^2^^,^^28^ Waning immunity leads to individuals becoming susceptible again, which highlights the necessity of booster vaccinations and enhanced surveillance in high-risk areas to prevent reinfection.

### Cholera responses

**Fig. 2:**
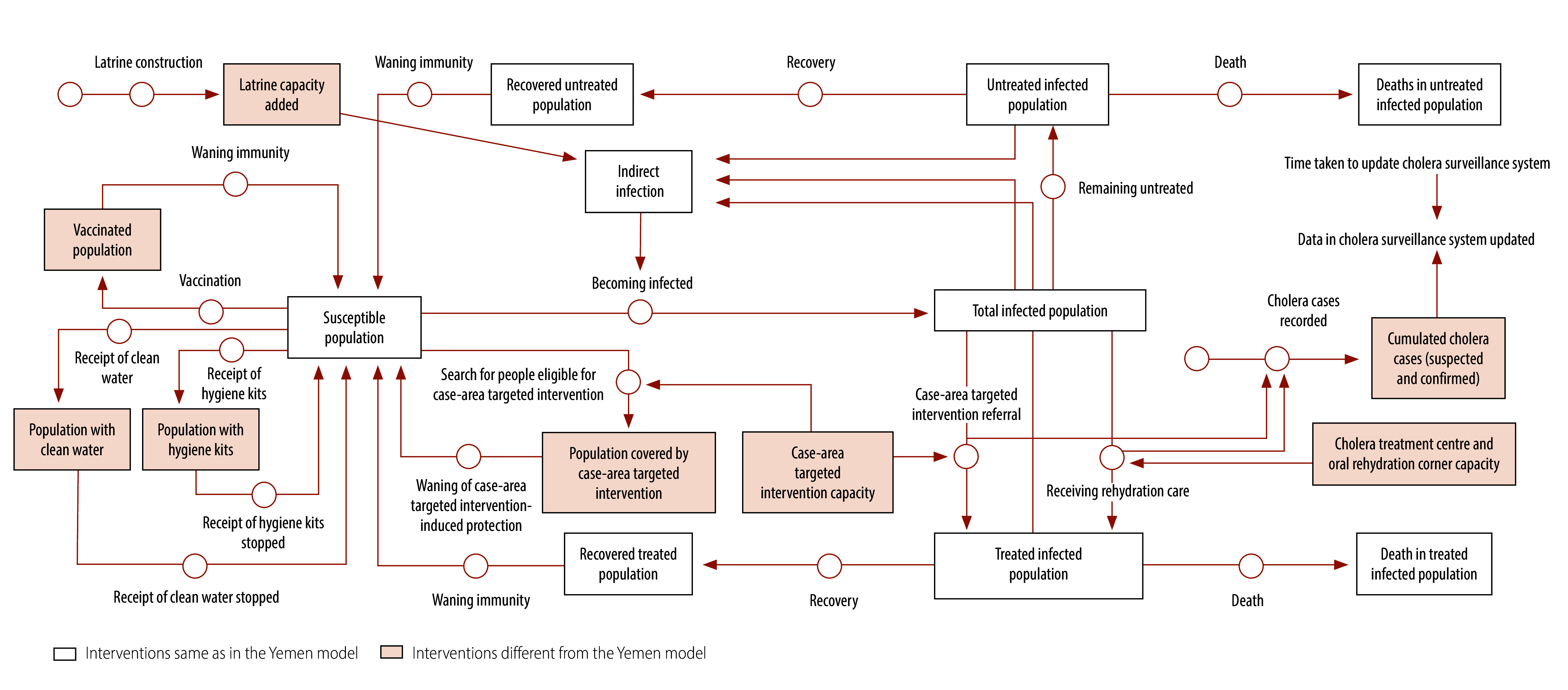
Simplified diagram of infection processes and interventions in the full cholera response model, modelling study of the effect of anticipatory action on cholera outbreaks in the Democratic Republic of the Congo

Notes: The cholera response model was based on a system dynamics model devised using data from a published model for Yemen.^16^ Indirect infection refers to the spread of cholera to susceptible individuals through contact with water that has been contaminated by individuals who are already infected. Oral rehydration corners were provided at community clinics.

Cholera response interventions are intended to reduce mortality and prevent disease transmission by integrating case management, water, sanitation and hygiene measures, vaccination, community engagement and surveillance.^11^^,^^29^ The cholera response model for the Democratic Republic of the Congo added case-area targeted interventions to the interventions used in the Yemen cholera response model to address the unique local context.^29^^,^^30^ Fig. 2 illustrates how these interventions were integrated into the susceptible–infected–recovered–susceptible framework. Box 1 summarizes the interventions included in the model (detailed descriptions are available in the online repository).^23^

Box 1 **Cholera response interventions, modelling study of the effect of anticipatory action on cholera outbreaks in the Democratic Republic of the Congo**

**Fixed chlorination points for clean water**

Chlorination points at community water sources to ensure drinking water is safe – an interim solution to reduce waterborne bacterial transmission^6^

**Hygiene kits**

Soap, oral rehydration salts and water purification tablets to address immediate household needs during cholera outbreaks^31^

**Latrine construction**

Sanitary latrines to prevent faecal contamination of water sources, thereby reducing the spread of cholera^32^

**Cholera treatment centres**

Inpatient facilities for severe cases, with sewage treatment systems to prevent environmental contamination^3^

**Oral rehydration corners**

Oral rehydration facilities at community-based clinics to prevent dehydration and reduce hospital admissions^33^

**Vaccination**

Oral cholera vaccine offering up to 70% protection after two doses and suitable for mass campaigns during outbreaks^34^^,^^35^

**Surveillance system**

Data on cholera cases collected from health centres to monitor the progression of the outbreak and guide response efforts^7^

**Case-area targeted interventions**

Rapid localized responses targeting areas within 100 to 250 m of a confirmed cholera case; responses may include water purification, sanitation measures, household visits, health education and active case-finding to quickly interrupt transmission in high-risk locations^29^^,^^30^

With anticipatory action, cholera outbreaks are mitigated proactively by mobilizing resources and implementing preventive measures before severe cases emerge.^9^^,^^10^ The activation threshold for anticipatory action was set by the United Nations Office for the Coordination of Humanitarian Affairs at 15 cholera cases per day (based on real-time surveillance data or reported case counts), and exceeding this threshold triggers the release of rapid action funds for intervention implementation.^36^ In our study, all interventions listed in Box 1 were regarded as being implemented once anticipatory action is triggered. This approach emphasizes preparedness and the efficient allocation of resources to minimize the impact of cholera on the affected population.^8^^,^^37^

### Model validation

Validation of our system dynamics model followed Barlas’s and Forrester & Senge’s guidelines,^38^^,^^39^ and included direct structure tests, behaviour tests and reproduction tests.^38^^,^^40^ A local sensitivity analysis, which involved varying parameters by plus or minus 15% over 100 runs using the Sobol sequence sampling method,^41^ identified 10 key parameters that influenced the model (Table 1). In addition, a global sensitivity analysis, which involved 100 000 simulations, further explored the effect of varying parameters by 15% (this range is commonly used for system dynamics validation).^38^^,^^42^

**Table 1:** Key parameters influencing the cholera response model, modelling study of the effect of anticipatory action on cholera outbreaks in the Democratic Republic of the Congo

Parameter^a^	Range for sensitivity analysis	Sensitivity^b^
Average duration of symptomatic illness, days	13.03–15.93	Numerical
Average incubation time, days	4.36–5.33	Numerical
Bacterial shedding from asymptomatic infected individuals, CFUs/person	81.62–99.76 × 10^3^	Numerical
Bacterial shedding from mildly infected individuals, CFUs/person	8.551–10.45 × 10^6^	Numerical
Bacterial shedding from severely infected individuals, CFUs/person	29.65–36.24 × 10^6^	Numerical
Contact rate with contaminated water, contacts/person per day	9.01–11.01	Numerical
Proportion of severely infected individuals seeking care	0.61–0.75	Numerical
No. individuals with severe cholera at time zero	1–3	Numerical
Time to contaminate water,^c^ days	17.17–20.98	Numerical
Disease transmission probability	0.05–0.06	Numerical

CFU: colony forming unit.

^a^ Parameters with the greatest influence on the cholera response model were identified in a sensitivity analysis, in which their values were varied by ± 15%.

^b^ Numerical sensitivity means that a variation in the parameter changes the magnitude of the model’s results (e.g. the number of cases) but does not alter the fundamental system behaviour.

^c^ The time to contaminate water is the assumed delay between the time when infected individuals shed *Vibrio cholerae* into the environment and the time when the pathogen reaches and meaningfully contaminates the water source (details are available in the data repository).^23^

**Fig. 3:**
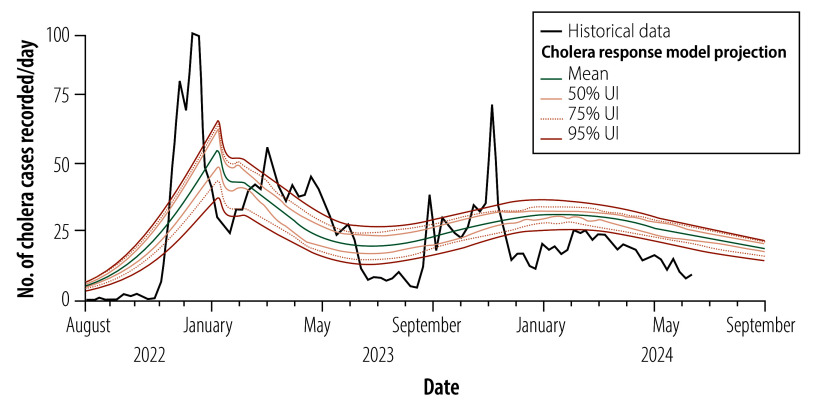
Comparison of historical cholera infection data and cholera response model projections, modelling study of the effect of anticipatory action on cholera outbreaks in the Democratic Republic of the Congo, August 2022 to September 2024

Fig. 3 compares historical data on the cholera infection rate in the Nyiragongo health zone during an outbreak between August 2022 and July 2024 with the corresponding projections of our cholera response model, including the results of a sensitivity analysis. Although the model’s projections align with the historical trend, there are some discrepancies. For example, the sharp increases observed in the historical infection rate in December 2022 and November 2023 are not replicated in the model’s projections, which reflects data inconsistencies and the exclusion of factors such as seasonality. Nonetheless, the model captures the dynamics of the cholera outbreak and the effect of the interventions, thereby enabling it to serve as a useful decision-support tool. The wide uncertainty intervals highlight the need for caution in interpreting the model’s projections. Details of the validation process are provided in the online repository.^23^

UI: uncertainty interval.

Notes: we obtained historical cholera infection data for an outbreak between August 2022 and July 2024 in Nyiragongo, Democratic Republic of the Congo. The cholera response model was based on a system dynamics model devised using data from a published model for Yemen.^16^

## Results

We evaluated the effect of four intervention scenarios on the dynamics of cholera infection in a hypothetical health zone in the Democratic Republic of the Congo between January 2022 and January 2025: scenario 1: baseline scenario corresponding to existing responses to a cholera outbreak; scenario 2: anticipatory action; scenario 3: anticipatory action plus one vaccine dose; and scenario 4: anticipatory action plus two vaccine doses. Table 2 summarizes the key features of these four scenarios, including differences in interventions, vaccination timelines and immunity periods.

**Table 2:** Intervention scenarios simulated using the cholera response model, modelling study of the effect of anticipatory action on cholera outbreaks in the Democratic Republic of the Congo

Scenario	Trigger for action	Interventions	Start of vaccination	Vaccination characteristics	Immunity period	Purpose
Scenario 1: baseline	Government outbreak declaration on 14 December 2022	All interventions in Box 1, excluding case-area targeted interventions	Mid-January 2023	280 000 doses delivered and 52% population coverage	6 months after single vaccine dose	To serve as a reference for other scenarios
Scenario 2: anticipatory action	Simulated case count reaches 15 per day on 25 October 2022	All interventions in Box 1	Mid-January 2023	280 000 doses delivered and 52% population coverage	6 months after single vaccine dose	To evaluate the impact of the early activation of interventions
Scenario 3: anticipatory action plus one vaccine dose	Simulated case count reaches 15 per day on 25 October 2022	All interventions in Box 1	One month after anticipatory action is triggered (i.e. November 2022)	280 000 doses delivered and 52% population coverage	6 months after single vaccine dose	To evaluate the combined impact of the early activation of interventions and a single vaccine dose
Scenario 4: anticipatory action plus two vaccine doses	Simulated case count reaches 15 per day on 25 October 2022	All interventions in Box 1	One month after anticipatory action is triggered (i.e. November 2022)^a^	280 000 doses delivered to 140 000 individuals and 26% population coverage	3 years after two vaccine doses	To evaluate the trade-off between the extended immunity provided by two vaccine doses and reduced vaccination coverage

^a^ The second vaccine dose was administered at least 7 days after the first.

**Fig. 4:**
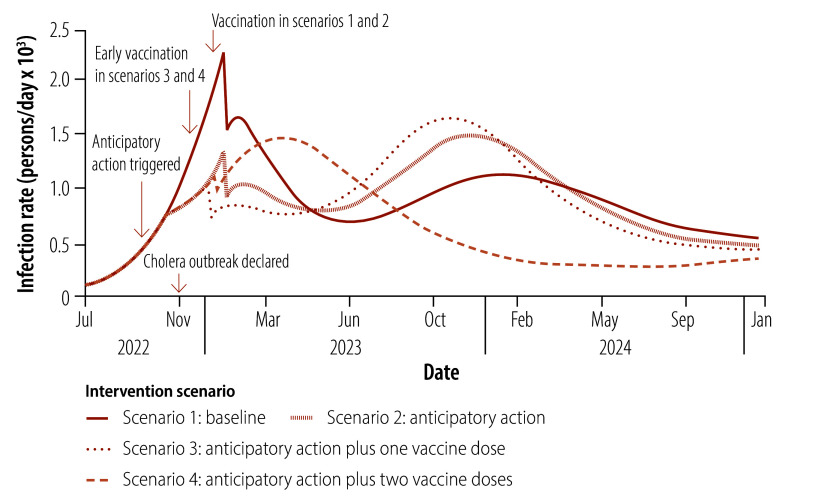
Daily cholera infection rates projected by the cholera response model for four intervention scenarios, study of the effect of anticipatory action on cholera outbreaks in the Democratic Republic of the Congo, July 2022 to January 2025

**Fig. 5:**
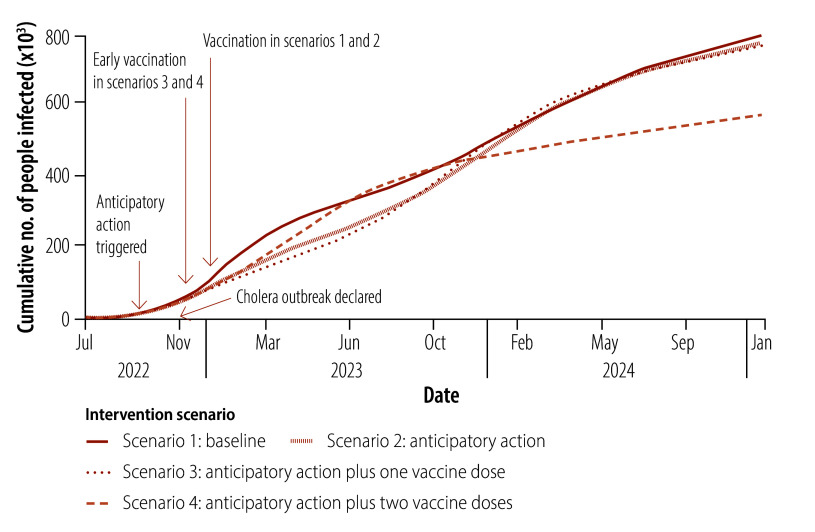
Cumulative cholera infections projected by the cholera response model for four intervention scenarios, study of the effect of anticipatory action on cholera outbreaks in the Democratic Republic of the Congo, July 2022 to January 2025

Fig. 4 and Fig. 5 illustrate the effect of these four scenarios on the cholera infection rate in the hypothetical health zone projected by our cholera response model. Asymptomatic cases are included. In addition, Table 3 lists the estimated cumulative infected population (both symptomatic and asymptomatic) 3, 6 and 15 months after anticipatory action was triggered in October 2022. These time-points reflect operational benchmarks commonly used in outbreak evaluations by humanitarian organizations, and highlight how the effect of the interventions evolves from the short to the long term. Box-and-whisker plots illustrating the long-term impact of each scenario are available in the online repository.^23^

**Table 3:** Total infected population projected by the cholera response model, by time and intervention scenario, modelling study of the effect of anticipatory action on cholera outbreaks in the Democratic Republic of the Congo

Scenario	Total infected population^a^							
	Time after anticipatory action was triggered in October 2022							
	3 months (January 2023)			6 months (April 2023)			15 months (January 2024)	
	Cumulative no.	Difference from baseline 1, %		Cumulative no.	Difference from baseline 1, %		Cumulative no.	Difference from baseline 1, %
1: baseline	141 417	Reference		266 349	Reference		500 513	Reference
2: anticipatory action	106 988	–24		192 832	–28		490 222	–2
3: anticipatory action plus one vaccine dose	100 649	–29		171 119	–36		508 134	2
4: anticipatory action plus two vaccine doses	105 192	–26		222 396	–17		455 617	–9

^a^ The total infected population includes both symptomatic and asymptomatic cases.

### Scenario 1

The baseline scenario models interventions that were implemented in the Nyiragongo health zone in 2022, excluding case-area targeted interventions, serves as a reference point. Fig. 4 shows that the number of infections rises consistently in this scenario to reach a peak in January 2023 when a vaccination campaign is launched to immunize 52% of the population of 540 000. This campaign markedly reduces cases by February 2023, which demonstrates that vaccination is effective in lowering disease transmission over the short term. However, the immunity conferred by the single vaccine dose wanes after 6 months and vaccinated individuals become susceptible again. Combined with gaps in vaccination coverage and unaddressed structural challenges, the waning of immunity creates a pool of vulnerable individuals. Seasonal factors and complacency about hygiene practices contribute to a second wave of infections by late 2023, which illustrates the limitations of relying solely on reactive interventions.

### Scenario 2

In this scenario, anticipatory action is triggered in October 2022 and interventions, such as case-area targeted interventions, the distribution of hygiene kits and latrine construction, are implemented immediately. These measures initially slow the spread of infection, which results in 24% (95% uncertainty interval, UI: 16–35) fewer infected individuals in January 2023, 3 months after anticipatory action was triggered, compared with the baseline scenario (Table 3). Despite this short-term success, infections peak again in January 2023 following cessation of the immediate interventions in December 2022 (Fig. 4). A subsequent vaccination campaign results in a temporary decline in the infection rate, mirroring the effect of a similar campaign in the baseline scenario. By late 2023, however, the absence of sustained measures leads to a resurgence in infections driven by the high number of susceptible individuals. This scenario highlights the vital need for sustained public health strategies to maintain infection control, such as ongoing hygiene education and booster vaccinations.

### Scenario 3

Scenario 3 features immediate interventions combined with early vaccination. As in scenario 2, anticipatory action triggered in October 2022 curbs the infection rate, which peaks in December 2022 before a rapid decline driven by early vaccine administration in late December (Fig. 4). By April 2023, 6 months after anticipatory action is triggered, there are 36% (95% UI: 25–51) fewer infected individuals than in the baseline scenario (Table 3), which demonstrates the efficacy of these combined strategies. However, the 6-month immunity period associated with the single vaccine dose results in a pronounced second wave, which peaks in November 2023. This resurgence in infection reflects immunity debt, which occurs when effective early vaccination enables people to avoid infection but limits the development of natural immunity. As the protection offered by the vaccine declines over time, a large portion of the population remains susceptible to infection. This scenario underscores the importance of complementary strategies, including booster doses, for sustaining long-term infection control.

### Scenario 4

In scenario 4, anticipatory action interventions are supplemented by a two-vaccine-dose strategy, with the second dose administered at least 7 days after the first. Our cholera response model assumes that 280 000 vaccine doses are used, as in scenario 3. However, the two-dose regimen results in a reduction in coverage to 26%, which limits the impact of vaccination on disease transmission, with infections initially peaking in January 2023 (Fig. 4). Nevertheless, the 3-year immunity period associated with two vaccine doses prevents a second wave of infection, which distinguishes this scenario from the other three. By January 2024, 15 months after anticipatory action is triggered, there are 9% (95% UI: 7–13) fewer infected individuals than in the baseline scenario (Table 3). Moreover, the cumulative total number of infected individuals over the long term is the lowest among all scenarios (Fig. 5). This result shows that there is a trade-off between vaccine coverage and immunity duration, and that strategic planning is important for maximizing the impact of vaccination in low-resource settings.

## Discussion

Our study used a modification of a published cholera response model to explore the effectiveness of anticipatory action against cholera outbreaks in a hypothetical health zone in the Democratic Republic of the Congo.^16^ Overall, we found that combining immediate and long-term interventions optimized disease outcomes, that booster vaccine doses are essential and that our cholera response model can serve as an aid to decision-making.

First, our analysis indicates that immediate interventions provide a critical foundation for long-term strategies, such as vaccination. Our cholera response model projected that triggering anticipatory action in October 2022 was associated with 24% fewer infected individuals at 3 months compared with the baseline scenario, which corresponds to around 35 000 fewer infections. This finding aligns with published studies,^29^^,^^43^ which highlighted the importance of rapid response measures for reducing transmission at the onset of outbreaks. However, a subsequent resurgence in infections was projected when only triggering anticipatory action, which underscores the limitations of standalone immediate interventions. This observation mirrors previous conclusions that sustained measures were needed to prevent new waves of infection.^44^ When we integrated immediate actions with a vaccination campaign, the projected number of infected individuals at 6 months was 64% of that in the baseline scenario. This synergistic effect demonstrates that immediate interventions can buy time for vaccination, thereby enhancing control efforts and extending their impact. Correspondingly, the Yemen cholera response model projected that starting interventions, particularly vaccination, sooner could avert 40% of deaths.^16^

Second, our analysis indicates that, to be effective, a single-vaccine-dose strategy in a resource-constrained context requires a subsequent booster dose. Our comparison of one-dose and two-dose vaccination strategies revealed an important trade-off. In scenario 3, the broad vaccine coverage achieved with a single dose is associated with 36% fewer infected individuals at 6 months than in the baseline scenario. However, waning immunity after 6 months leads to a resurgence in infections, which highlights the phenomenon of immunity debt. Consequently, as concluded by others, booster campaigns are important for sustaining immunity.^13^^,^^45^

In our analysis, the projected cumulative number of people infected by January 2025 was approximately 30% lower when immediate interventions were combined with a two-vaccine-dose strategy, than with either the baseline scenario, scenario 2 or scenario 3. Despite this benefit, the lower vaccine coverage in scenario 4 means that 74% of people would not be covered, thereby leaving a larger proportion of the population susceptible to infection. This observation underscores the challenge of balancing immunity duration and vaccination coverage in resource-constrained settings. Findings from published studies suggest that single-dose vaccination campaigns may be more effective during outbreaks when vaccine supplies are limited. ^45^^,^^46^ Nevertheless, long-term protection requires strategic planning for booster doses.

Third, in addition to its role in evaluating interventions, our cholera response model can serve as a dynamic platform for decision-making. By simulating outbreak scenarios, the model enables policy-makers to evaluate intervention strategies in a risk-free environment and to conduct real-time adjustments based on the model’s projections. Other researchers have also found that interactive simulations were valuable for public health planning.^47^^,^^48^

We found that water, sanitation and hygiene interventions play a crucial role in the management of cholera outbreaks over the long term. Emergency measures, such as fixed chlorination points, offer immediate benefits but coverage may be limited, as observed in Malawi and Yemen.^26^^,^^49^ The Global Task Force on Cholera Control’s 2030 roadmap stresses that long-term investment in water and sanitation infrastructure is essential to progress from emergency responses to sustainable public health.^11^ Although our focus was on medium-term interventions, our findings underscore the need for comprehensive improvements in water, sanitation and hygiene services alongside vaccination and immediate actions in response to disease outbreaks.

The results of our model indicate that triggering predefined anticipatory action during cholera outbreaks can improve outcomes compared with conventional outbreak declarations that occur weeks later. Health authorities are thus provided with a practical strategy for acting earlier, which could accelerate the release of funding and the roll-out of interventions. In settings such as North Kivu, this could translate into earlier vaccination (i.e. November 2022 rather than mid-January 2023 in our baseline scenario) and the faster deployment of hygiene measures and case-area targeted interventions – actions that national ministries, humanitarian organizations and their partners could adopt immediately if thresholds for anticipatory action were embedded in existing surveillance protocols.

To simplify the analysis, our cholera response model did not consider population displacement, conflict or seasonal factors but focused instead on broader population dynamics. Consequently, key drivers of cholera among refugees and internally displaced persons were omitted. Future work should include these drivers and stratify the population by age and gender. Moreover, as the ranges of some key parameters were hypothetical, it is important that these ranges are continuously calibrated with field data on the performance of interventions and with updated epidemiological information to strengthen confidence in the model’s projections. Applying the model to other cholera hotspots will require adaptations to region-specific circumstances, and iterative validation in partnership with local stakeholders so that differences in water, sanitation and hygiene infrastructure, health-care capacity and security constraints are considered.

In conclusion, our study demonstrates that anticipatory action can enhance cholera outbreak management in low-resource settings, such as the Democratic Republic of the Congo, by combining immediate interventions with vaccination. Triggers for early anticipatory action that initiate interventions weeks before traditional outbreak declarations can buy critical time for deploying vaccines and for implementing other disease control measures, thereby reducing the risk of immunity debt and subsequent waves of infection. Our adapted cholera response model can serve as a dynamic learning platform that enables decision-makers to simulate and refine intervention strategies in a risk-free environment, thus supporting a shift from reactive to proactive planning in line with the 2030 Global Task Force on Cholera Control’s roadmap. Future work on the model should explore its application to other regions with a high burden of disease, the use of real-world data for calibration and validation, and the incorporation of contextual factors such as population displacement. Ongoing refinement will strengthen the model’s utility as a tool for public health decision-making and global efforts to control and eliminate cholera.
